# Cultural foundations of global health: a critical examination of universal child feeding recommendations

**DOI:** 10.1186/s41256-025-00405-1

**Published:** 2025-01-23

**Authors:** Gabriel Scheidecker, Leberecht Funk, Nandita Chaudhary, Bambi L. Chapin, Wiebke J. Schmidt, Christine El Ouardani

**Affiliations:** 1 University of Zurich, Zurich, Switzerland; 2 Hamburg, Germany; 3 University of Delhi, New Delhi, India & Universidade da Bahia, Salvador da Bahia, Brazil; 4 University of Maryland, Baltimore County, USA; 5 Osnabrück University, Osnabrück, Germany; 6 California State University, Long Beach, USA

**Keywords:** Child feeding recommendations, Early childhood, Decolonization, Epistemic injustice, Ethnographic research, Cultural diversity

## Abstract

There has been a rising call to decolonize global health so that it more fully includes the concerns, knowledge, and research from people all over the world. This endeavor can only succeed, we argue, if we also recognize that much of established global health doctrine is rooted in Euro-American beliefs, values, and practice rather than being culturally neutral. This paper examines the cultural biases of child feeding recommendations as a case in point. We argue that the global promotion of Responsive Feeding—a set of allegedly best practices for child feeding promulgated by the WHO and others—is based on a tacit conviction that certain Western middle-class feeding practices are universally best, along with a promise that future evidence will demonstrate their superiority. These recommendations denounce feeding practices that diverge from this style as Non-Responsive Feeding, thereby pathologizing the many valued ways of feeding children in communities all over the world without sound scientific evidence. Drawing on ethnographic research, we show that there is a wide variety in feeding practices around the world and these are closely interlinked with the understandings and priorities of caregivers, as well as with favored forms of relationships and ways of maintaining them. For global health nutrition interventions to be justified and effective, they would need to be based on more pertinent, culturally responsive research than they currently are. We suggest the use of ethnographic research as an important tool in building empirically grounded, epistemically inclusive, and locally meaningful approaches to improving nutritional support for children in communities around the world and to global health efforts more broadly.

## Background

Global health science is dominated by Euro-American institutions and perspectives, while research, expertise, and knowledge from the Majority World, or so-called low- and middle-income countries, is often excluded or framed as inferior [1–3]. A crucial step in decolonizing global health must consist of ending this kind of epistemic injustice [4] and exclusion [5], and building a knowledge base that includes diverse perspectives and research from around the world [6]. Drawing on such insights, researchers in the field of global health have increasingly recognized the need to consider the knowledge, values, and practices of targeted communities and families as an important cultural foundation for intervention [7]. However, much less consideration has been given to critically interrogating the hegemonic, Western middle-class culture of the dominant researchers and research institutions that co-determine global health science and practice, which often leads to cultural biases influencing interventions that end up being unhelpful or even harmful.

We argue that starting with local knowledge, practices, and concerns as a basis for global health intervention and turning a critical eye towards the Western cultural foundation of scientific knowledge production are both needed to work effectively towards epistemic inclusion and the decolonization of global health. Scrutinizing the cultural biases of existing scientific claims in global health is crucial to counteract the imposition of Western cultural preferences under the guise of scientific evidence and to open a space for epistemic inclusion. To make use of this space and to create a more comprehensive, globally balanced knowledge base for a culturally relevant global health, it is necessary to research culturally grounded knowledge and practices around the world.

Responsive Feeding interventions that aim to change the way caregivers feed their children illustrate these concerns in a particularly stark fashion. Responsive Feeding (RF) refers to a set of allegedly evidence-based standards for the feeding interaction between caregivers and young children. Inspired by the principle of sensitive responsiveness promulgated by attachment theory, RF emphasizes that caregivers should follow the child’s lead by responding sensitively to their cues of hunger and satiety [8–12]. Furthermore, caregivers are advised to interact face-to-face with children while feeding, to respond to them by smiling and praising, to be attentive and patient, and to avoid distractions. Caregivers should also encourage early self-feeding by providing finger foods. A basic assumption of RF is that eating is an individual, autonomous activity in which caregivers only play a transitory and assisting role.

Since the early 2000s, RF has been rapidly incorporated into global health guidelines and policies with the goal of reducing early childhood malnutrition by training parents in this feeding style [9]. Significantly, RF recommendations were included in the major WHO and UNICEF guidelines for child feeding in 2000 [13] and 2003 [14]. Despite the inclusion of these recommendations, most of the research on RF intervention effects in the Majority World was only conducted in the following decade and systematically reviewed in 2011 [15]. This research has failed to provide clear evidence of the benefits of RF interventions, as we show below; nevertheless, global health guidelines and policies continue to recommend RF as an evidence-based solution to malnutrition. Most prominently, RF is currently being promoted through the Nurturing Care Framework, a roadmap for the systematic global implementation of early childhood interventions that was launched jointly by the WHO, UNICEF, and the World Bank and accepted by the World Health Assembly in 2018 [16]. A recent Nurturing Care thematic brief dedicated to RF states that RF interventions “should be implemented for all children everywhere” (p. 13). [17]

In this article, we take the inclusion of RF recommendations into the Nurturing Care Framework as an opportunity to review the underlying research and potential cultural biases. We first demonstrate that the evidence for the global promotion of RF as an allegedly optimal form of care is neither scientifically objective nor comprehensive but anchored in Western middle-class culture and tacit belief in the superiority of this culture. Second, we show that existing ethnographic evidence about feeding practices around the world contradicts the claim that RF is a universally optimal care practice. Rather, feeding practices vary as a function of diverse food cultures, parenting styles, and ways of building and expressing social relationships. We argue further that ethnographic approaches are useful to work towards epistemic inclusion—meaning the inclusion of knowledge about various forms of child feeding across diverse cultural contexts—in research and policy. The careful scrutiny of potential cultural biases in existing global health science, along with the integration of the knowledge and skills of targeted communities, is crucial to achieving epistemic decolonization. Building a comprehensive knowledge base will help support both children’s health and the strength of their communities.

## The shaky evidence for the benefits of responsive feeding

Research publications and policy documents commonly justify RF interventions as being based on rigorous scientific evidence. In this section, we investigate the evidence that they cite in support of RF feeding interventions. We begin with research that claims to be universally valid and move on to research pertaining more specifically to low- and middle-income countries.

A recent article that promotes the expansion of RF guidelines within the *Nurturing Care Framework* [10] states:

*Consistent evidence* from randomized controlled trials indicates that providing RF guidance to mothers on how to recognize and respond appropriately to children's hunger and satiety cues can lead to improved weight status among infants and young toddlers. (p. 2, emphasis added)

To support this claim, the article cites the most recent scientific review on RF interventions [11], which concludes:

*Moderate evidence* from randomized controlled trials suggests that providing responsive feeding guidance to mothers to recognize and respond appropriately to a child’s hunger and satiety cues can contribute to “normal” weight gain and/or “normal” weight status in children 2 y and younger compared with children whose mothers did not receive responsive feeding guidance. (p. 990S, emphasis added)

“Consistent evidence” thus turns out to be “moderate evidence”. But even the estimation of “moderate” appears to be generous when taking a closer look at the specific results of the review. Of the four randomized controlled trials reviewed, two were effective at reducing or preventing childhood overweight, while one found only a temporary effect and another was found to have limited validity. However, the three valid trials also included other components in the experimental conditions such as “increased exposure to healthy foods and decreased exposure to unhealthy foods” (p. 994S) [11]. This makes it impossible to know whether the RF component was, in fact, responsible for the measured decrease in children’s overweight.

Several longitudinal cohort studies were also reviewed and equally judged to provide “moderate evidence” [11]. While pointing to an association of “restrictive feeding practices” with increased weight gain and “pressuring feeding practices” with decreased weight gain, they did not examine the direction of effect. That is, they could not rule out that these so-called non-responsive feeding practices may have been a response to children’s weight status or appetite rather than the cause. In fact, another study which specifically examined the direction of these effects found that “Maternal feeding practices become more controlling after and not before excessive rates of weight gain” [18].

Not only is this evidence in the review more weak than moderate, it is not representative of the global population. Among a total of 27 studies that were examined [11], 17 were from the USA, four were from Australia, four were from the UK, one was from the Netherlands, and one was from China. The Chinese study “did not find any association between responsive feeding practices and change in BMIZ” [11]. All four randomized controlled trials were conducted in the US or Australia, and the three which showed effects “were conducted in mothers who were mostly white, middle-class, and college-educated” [11]. Thus, the “moderate evidence” for the benefits of RF is overwhelmingly based on Western middle-class research participants who have been shown to poorly represent the global population [19].

A second reason why the findings from these RF studies in high-income countries cannot be generalized is that they focus largely on the reduction or prevention of overweight, while RF interventions in low- and middle-income countries focus on the reduction of underweight or stunting. Even if there were clear evidence that RF interventions help reducing overweight, which is not the case, this would not allow the conclusion that such interventions are also effective in reducing undernutrition.

While studies on RF interventions to reduce undernutrition in low- and middle-income countries do exist, they are even rarer. The above-mentioned *Nurturing Care* thematic brief [17], for example, refers to only one study in support of the claim that RF interventions reduce malnutrition in the Majority World. This study conducted by Vazir et al. in India [12] compares a control group with two intervention groups: one that received complementary food recommendations and one that received these recommendations plus trainings in both RF and psychosocial stimulation. While both intervention groups showed improved nutritional intake, only the non-RF intervention group reduced stunting. These results suggest that the RF training had no independent impact on food intake and that RF or psychosocial training even counteracted the reduction of stunting found in the non-RF intervention group. This study indicates, in fact, that RF interventions may be harmful even though the results are being used in the Nurturing Care thematic brief to argue for universal implementation of these interventions.

Scant and conflicting evidence is not just an issue in the Nurturing Care thematic brief. An article [20] from the Lancet series that has provided the evidence for the Nurturing Care Framework, bases the inclusion of RF just on two studies: the above trial by Vazir et al. [12] and another study [21] conducted by Aboud and colleagues in Bangladesh. While the first study did not show an independent, positive effect of the RF intervention, as demonstrated above, the second study by Aboud and colleagues did so. To put this finding of an individual study into perspective, we may refer to a scientific review of 21 RF studies in low- and middle-income countries [15]. This review concludes that “overall, few studies have demonstrated a positive association between RF and child undernutrition” (p. 502). Of the 21 reviewed studies, 19 combined RF with other intervention components making it impossible to identify the independent RF effects.

Of the 2 interventions explicitly designed to manipulate RF, results were mixed, with 1 study showing significantly greater weight gains and attained weights among intervention compared with control children [21], but the other found no significant differences between groups [22] (p. 505).

The first study with positive results is the one by Aboud and colleagues in Bangladesh, which the Nurturing Care Lancet article cites. The second study without positive results, however, is not cited in the Lancet article. Neither the Lancet article nor the thematic brief cite the review about RF interventions in low- and middle-income countries although its purpose is to provide a more comprehensive evidence base than individual, cherry-picked studies.

Even though more than two decades have passed since the incorporation of RF into global health policies, the promise of the post hoc scientific legitimation of RF interventions as a remedy for malnutrition remains unfulfilled. The Nurturing Care thematic brief tries to keep the hope for future scientific evidence alive by saying that “more evidence from low-income countries [on the association between RF and malnutrition/underweight] is needed.” [17] Simultaneously, it raises a new hope that, beyond nutritional benefits, “responsive feeding may help improve psycho-emotional and cognitive development” (p. 4) [17]. Clearly, RF interventions are not based on existing scientific evidence but rather on the promise of prospective scientific evidence. If advocates insist on the benefits of RF interventions by persistently pointing to future evidence or proposing new hypothetical benefits, we must assume additional, extra-scientific reasons for keeping such interventions in place. We suppose that an important reason for promoting RF interventions is a tacit view that RF principles must be good practice because they originate from Western middle-class culture.

## Responsive feeding as a Euro-American middle-class concept

Responsive feeding is a Western middle-class concept. First, the theoretical framework of RF, which is attachment theory, largely emerged from research and theorizing in British and US settings before it was globally applied [23, 24]. Second, many cross-cultural studies have shown that the reciprocal, quasi-equal interaction style of RF, including the emphasis on frequent eye contact, verbalization, and praise, is typically endorsed by Western middle-class families but not necessarily by people from the Majority World [25]. Third, the central idea of RF—that the autonomy of children needs to be respected by responding readily to their emotional expressions and granting them a leading role in the interaction—is grounded in a specific Western, middle-class, neoliberal, individualistic conception of the self. It is one of the most consistent findings in anthropology and cross-cultural psychology that societies and communities in the Majority World foster different self-construals, often with an emphasis on interdependency and relatedness [26, 27].

The fact that only Western feeding practices have been considered and tested as a global remedy for malnutrition, while equally long-standing feeding practices from Majority World cultural contexts have been dismissed or ignored, suggests that there exists an inherent assumption of Western superiority amongst its advocates. Even if there was clear evidence that RF enhances the nutritional state of children—which is not the case—other established feeding practices from the Majority World could potentially lead to the same or better outcomes. Yet this possibility is ignored. This is in spite of alternative approaches to children’s nutrition that have been demonstrated to work successfully by amplifying local skills rather than imposing external standards [28].

The ongoing promotion of RF over the last 20 years as a solution to malnutrition, despite the absence of compelling scientific evidence, suggests that the belief in Western superiority is a driving force. Therefore, the dictum of the Nurturing Care thematic brief, “more research is needed,” may instead be taken to mean: “Since RF is from the West, it must be best, and we just need to work harder to prove it.” Supremacism manifests itself not only in the-taken-for-granted belief in the superiority of Western middle-class practices, but also the assumed inferiority of non-Western practices.

## The devaluation of majority world feeding practices

The RF literature conceptualizes feeding practices in the Majority World largely in negative terms. All feeding practices that do not correspond to the principles of RF are labeled as Non-Responsive Feeding (NRF). They are thus measured by an external standard and defined only by deviation from that standard. Given the fact that RF reflects Western middle-class understandings and preferences about proper parent–child interaction, it is no surprise that the feeding practices of Majority World families are mostly classified as NRF. A study conducted in Ghana, for example, reports that 81.2% of Ghanaian parents used NRF [29].

An approach that classifies feeding practices from around the world solely on the basis of their deviation from an external, Western-influenced standard undermines any attempt to understand Majority World practices on their own terms and to recognize their potential benefits for children. It also ignores the diverse meanings and purposes caregivers attach to their feeding practices, as well as the adaptive value such practices may have in specific socioeconomic environments. In short, to classify the practices of Majority World parents as NRF is to deny these parents a position as knowing and skilled caregivers. In our view, this is a clear case of epistemic exclusion [5] and a confusion of cultural difference with deficiency [30].

In conjunction with epistemic exclusion, the RF literature engages in the devaluation of Majority World feeding practices. According to the above-mentioned Nurturing Care thematic brief [17], a caregiver who does not follow the principles of RF “dominates the feeding situation,” “ignores the child,” or “fails to direct child behaviours that interfere with the establishment of healthy food preferences and eating routines” (p. 3). In a more subtle way, the thematic brief even implies that NRF is associated with a lack of parental love, stating that a “patient and loving caregiver” follows the principles of RF (p. 2). Taken together, the RF literature systematically devalues Majority World parents by presenting them as non-responsive child feeders who are lacking knowledge, skills, sensibilities, and even love for their children.

RF advocates may see the devaluation of Majority World caregivers as mere collateral damage on a mission to reduce malnutrition in children and to train these caregivers in “respecting the autonomy of the child” through RF (p. 4) [17]. However, as we have demonstrated in the previous section, the evidence that RF improves children’s nutritional status is shaky, not only for the Majority World but also for the Minority World, and the depiction of all other practices as suboptimal is entirely unfounded. Hence, rather than a side effect, the devaluation of caregivers in RF research and intervention may simply be the main effect. Echoing a recent reminder [31], we insist that global health research and practice must respect the dignity and autonomy of the targeted people, including the caregivers of children.

## Epistemic inclusion: a crucial step towards decolonizing global health

Taking the knowledge, skills, and priorities of caregivers seriously when developing and implementing child-focused support is, in our view, imperative for ensuring that the autonomy of targeted families and communities is respected. In addition, respecting caregivers as knowledgeable agents is a precondition for providing effective and meaningful support [32]. For both reasons, epistemic inclusion represents a crucial step towards decolonizing global health.

Existing ethnographic research is a good starting point for pursuing this objective. Ethnographic research has been designed to avoid the imposition of external standards rooted in Western cultural norms. This method aims to maximize sensitivity to the knowledge and practices of research populations and to their specific social, cultural, and economic contexts. To achieve this goal, ethnographic research employs long-term participant observation so that researchers gain in-depth understanding of the research population and build trusting relationships, along with additional qualitative and quantitative methods that are carefully aligned with the local conditions. Unfortunately, in the field of global health and especially in the domain of early childhood intervention, the extensive body of ethnographic research about early childhood across cultural contexts remains largely untapped [33]. To showcase this potential, we offer four implications from ethnographic research for RF interventions based on our recent comparative examination of feeding practices in Morocco, Madagascar, Sri Lanka, Taiwan, and Costa Rica: [34]

1. Feeding practices vary considerably across the world in accordance with food cultures and parenting styles. The caregivers in the five groups we studied offer a sample of this diversity. In four groups, the ways caregivers fed their children departed significantly from the principles of RF. However, this was not due to a lack of responsiveness or ignorance among caregivers but rather because of their specific, culturally embedded understandings and values. As other research shows as well, practices related to food and feeding are an intimate and valued dimension of society [35]. Feeding practices are also integrated with other parenting practices and socialization goals [36]. Thus, direct interventions in feeding practices do not just affect the practices themselves, but likely have other effects on cultural and interpersonal dynamics.
2. Feeding practices are embedded in and contribute to valued forms of social relationships. In four of the five groups, feeding practices were closely associated with the formation of hierarchically organized relationships and corresponding emotions. In cross-cultural research, it is well-established that models of social relationships, kinship, self, and emotion vary considerably across social groups [26, 27]. While RF interventions are increasingly promoted by their advocates as improving socio-emotional development, they may, in fact, simply change social relationships, shifting them towards a more individualistic model. Among other effects, this may undermine intergenerational support and cohesion, something which is particularly crucial where state support is weak or not accessible.
3. Feeding is understood as an important expression of care and affection across socio-cultural settings, but the ways in which these emotions are expressed through feeding interactions vary considerably. While RF locates the expression of positive emotions exclusively in a feeding style defined by sensitive responsivity, eye-contact, smiling, and verbal expressions, several of the groups we studied view the provision of food or the act of feeding itself as a crucial expression of care and love. The prioritization of acts of care as demonstrations of affection over verbal or facial expressions has been documented in many non-Western societies [37]. Interventions that only recognize one form of affection as legitimate may disrupt more deeply felt and culturally-salient enactments of attachment and care.
4. The age at which children begin to eat independently varies considerably across groups—not as a function of parenting skills, but rather because of the specific socio-emotional role of feeding in a particular context. In the Sri Lankan group, for example, caregivers continued to hand-feed their school-aged children because they considered it both practical and an important way to express affection and maintain close relationships. RF recommendations, by contrast, aim to promote early independent eating and frame the direct feeding of older children as NRF. Since no evidence for the benefits of early independent eating is presented anywhere in the literature, we must assume that early independent eating is as much rooted in culturally specific concepts and values as prolonged hand-feeding is. Insisting on early and exclusive self-feeding, as RF does, works to eliminate a wide range of experiences that may socialize children into valued forms of relationships and configurations of self.

## Conclusions

Our analysis has shown that RF recommendations, contrary to the claims of their proponents, do not rely on solid scientific evidence. Instead, we have argued that such recommendations and the research on which they are based are strongly influenced by the hegemonic Western middle-class culture from which they have predominantly emerged. Feeding practices originating from the Majority World have neither been considered for recommendation nor researched through intervention trials. This omission occurred despite the fact that feeding practices are usually well adapted to the local conditions, as ethnographic research shows. We suggest that it may be a sense of cultural superiority that explains why RF proponents continue to recommend RF interventions as a remedy to malnutrition in the Majority World, despite the lack of solid scientific evidence. Furthermore, we have demonstrated that ethnographic research in diverse settings in the Majority World is crucial to identify cultural biases in existing global health claims. Apart from its deconstructive role—which we believe is necessary to create the space for epistemic inclusion—ethnographic research may also play a constructive role. By focusing on specific local circumstances, practices, and knowledge, ethnography can help to align global health programs with the goals, needs, and strengths of parents, families, and communities. For this purpose, existing ethnographic evidence needs to be complemented by new ethnographic research when planning interventions designed to support families. Crucially, such formative research should not just pursue the question of how to improve the delivery of behavioral-change interventions that are based on predefined standards like RF. It should also determine *what* kind of support caregivers actually need and see as helpful, since this is the only way to respect them as knowledgeable and skilled agents. Some may see it as a tedious and costly endeavor to consider caregiver input and to use these insights to guide local interventions. However, large-scale studies designed to provide post-hoc evidence for already-implemented programs may use up even more time and resources, especially if interventions turn out to be ineffective or even harmful. The use of ethnographic research will not only help to align nutritional support with local conditions, but it will also contribute to globally inclusive knowledge production, making a concrete contribution towards decolonizing global health.

## Data Availability

Not applicable.
